# Survival Outcomes in Stage IV Small Bowel Neuroendocrine Tumor Patients Undergoing Loco-Regional Surgery: An Experience From a Pakistani Cancer Center

**DOI:** 10.7759/cureus.81259

**Published:** 2025-03-26

**Authors:** Zain Tayyab, Taha Ahmad, Muhammad Zubair, Sana Hussain, Talha Javed, Adnan Khalid, Hadia Munir Baig, Noor Fatima Chaudhry, Talha Bin Nasir, Nain Tara

**Affiliations:** 1 Surgical Oncology, Shaukat Khanum Memorial Cancer Hospital and Research Centre, Lahore, PAK; 2 General Surgery, Shaukat Khanum Memorial Cancer Hospital and Research Centre, Lahore, PAK; 3 General Surgery, Combined Military Hospital (CMH) Lahore Medical College and Institute of Dentistry, Lahore, PAK; 4 Community Medicine, Al-Aleem Medical College, Lahore, PAK; 5 Physiology, Lahore Medical and Dental College, Lahore, PAK; 6 Internal Medicine, Guthrie Robert Packer Hospital, Pennsylvania, USA

**Keywords:** loco-regional resection, lower-middle-income countries (lmics), neuroendocrine tumor management, oncologic surgery, small bowel neuroendocrine tumors (sb-nets), survival outcomes

## Abstract

Background: Small bowel neuroendocrine tumors (SB-NETs) are rare malignancies often diagnosed at an advanced stage due to their nonspecific symptoms. The role of loco-regional surgery in stage IV SB-NETs remains debated, particularly in lower-middle-income countries, where healthcare resources and treatment accessibility differ from high-income settings.

Methods: This retrospective study analyzed survival outcomes in stage IV SB-NET patients who underwent loco-regional resection at Shaukat Khanum Memorial Cancer Hospital & Research Centre between 2014 and 2023. Patients with one to three hepatic metastases who underwent surgical resection were included. Clinical, pathological, and survival data were reviewed. Median survival at five and ten years was assessed.

Results: Fifteen patients met the inclusion criteria. The median age was 47 years, with abdominal pain in all 15 patients (100%). Other symptoms included weight loss in four patients (26.7%), vomiting in four patients (26.7%), and carcinoid symptoms in three patients (20%). The ileum was the most frequently affected site in seven patients (46.7%), followed by the jejunum in four patients (26.7%). Surgical resection included small bowel resection in nine patients (60%) and hepatic metastasectomy in all 15 patients (100%). Adjuvant therapy was administered to all patients. The 30-day and 90-day mortality rates were one (6.7%) and two (13.3%), respectively. The median survival was 60% at five years and 33.3% at 10 years.

Conclusion: These findings suggest that surgical intervention may not offer a significant survival advantage. While our findings are consistent with existing literature regarding the predominance of small bowel NETs and the role of surgery in treatment, the small sample size and single-institution nature of this study limit the generalizability of our results.

## Introduction

Small bowel neuroendocrine tumors (SB-NETs) are rare malignancies originating from serotonin-producing enterochromaffin cells of the gastrointestinal tract [1]. They have an estimated incidence of 3 to 5 per 100,000 individuals [2]. The clinical presentation of SB-NETs varies, with some patients experiencing nonspecific symptoms such as abdominal pain, diarrhea, and weight loss, while others develop classic carcinoid syndrome, characterized by flushing, bronchospasm, and severe secretory diarrhea [3]. These tumors often remain asymptomatic until they metastasize, leading to a high proportion of cases being diagnosed at stage IV, with significant mesenteric lymph node and hepatic involvement [4]. Despite their metastatic nature, SB-NETs have a relatively favorable prognosis compared to small bowel adenocarcinomas, with reported five-year survival rates between 61% and 77% [5].

Surgical resection is the standard of care for localized SB-NETs; however, its role in patients with stage IV disease remains controversial [6]. Loco-regional resection of the primary tumor is performed to prevent complications such as bowel obstruction, ischemia, and severe carcinoid syndrome [7]. Some studies suggest that debulking surgery may improve survival in select patients with limited hepatic metastases, but robust comparative data remain lacking [8]. Most available literature on SB-NET treatment comes from high-income countries, where access to advanced multidisciplinary care, systemic therapy, and surgical expertise differs from that in lower-middle-income countries (LMICs) [9].

This study provides a retrospective analysis of our experience with survival outcomes in stage IV SB-NET patients who underwent loco-regional resection at Shaukat Khanum Memorial Cancer Hospital & Research Centre (SKMCH), a dedicated cancer center in an LMIC. While this study does not include a control group of non-surgically managed patients, it provides valuable survival data for a population where healthcare resources and treatment accessibility may differ from those in high-income settings. The aim of this study is to evaluate the impact of loco-regional surgery on survival outcomes in patients with stage IV SB-NETs with limited hepatic metastases, within the context of an LMIC.

## Materials and methods

Study design

We retrospectively reviewed our institutional neuroendocrine tumor database to identify all patients diagnosed with stage IV SB-NETs who underwent loco-regional resection at Shaukat Khanum Memorial Cancer Hospital & Research Centre (SKMCH) from 2014 to 2023. The study was approved by the Institutional Review Board (approval number EX-03-02-25-04) and was conducted in accordance with the Declaration of Helsinki.

Data collection

Patient data were obtained from the institutional neuroendocrine tumor database and electronic medical records. Information collected included demographic details, clinical presentation, tumor characteristics, operative details, postoperative course, and survival outcomes. Any postoperative complications or readmissions were also documented to evaluate the impact of loco-regional resection on patient outcomes.

Patient selection

A total of 70 patients diagnosed with stage IV SB-NETs were reviewed. Patients were categorized based on the presence of hepatic metastases and whether they had undergone loco-regional resection of the primary tumor. The inclusion criteria comprised patients with stage IV SB-NETs who had 1 to 3 hepatic metastases and underwent loco-regional resection. Patients were excluded if they had dual malignancies, chronic kidney disease, chronic liver disease, congestive cardiac failure, or more than three hepatic metastases. Based on these criteria, 15 patients were included in the final analysis.

Variables analyzed

Several clinical and pathological variables were analyzed to assess their impact on patient outcomes. Demographic variables included age and gender, while clinical presentation variables encompassed symptoms such as abdominal pain, diarrhea, flushing, weight loss, and bowel obstruction [10]. Tumor characteristics were evaluated based on the primary tumor site, size (median 1.5 cm), and histological differentiation. The Ki-67 proliferation index, an important marker of tumor aggressiveness, was recorded to classify tumors based on their proliferative activity [11]. Treatment-related variables included the extent of primary tumor resection, hepatic metastasectomy, and the use of adjuvant therapy such as somatostatin analogs or chemotherapy [12]. Survival outcomes were assessed using overall percentage survival at five and 10 years and postoperative mortality rates at 30 and 90 days.

Statistical analysis

Data was analyzed using IBM SPSS Statistics for Windows, Version 20.0 (Released 2011; IBM Corp., Armonk, NY, USA) with median survival rates calculated for five-year and 10-year follow-ups.

## Results

A total of 70 patients diagnosed with stage IV SB-NETs were reviewed. Among them, twenty-one patients had hepatic metastases, and 15 patients met the inclusion criteria for loco-regional resection and were included in the final analysis. The median age of the patients was 47 years.

The most common presenting symptoms are shown in Table 1. Abdominal pain was present in all 15 patients (100%), making it the most frequently reported symptom. Other common symptoms included weight loss in four patients (26.7%), vomiting in four patients (26.7%), and carcinoid symptoms in three patients (20%). A subset of two patients experienced diarrhea and melena (13.3%), while jaundice was present in one patient (6.7%) and dyspepsia in one patient (6.7%). 

**Table 1:** Clinical Presentation of Patients with Stage IV SB-NETs (n=15) SB-NETs: Small bowel neuroendocrine tumors

Clinical Feature	Number of Patients (n=15)	Percentage (%)
Abdominal Pain	15	100%
Diarrhea and Abdominal Pain	2	13.3%
Abdominal Pain, Melena, and Diarrhea	2	13.3%
Dyspepsia	1	6.7%
Weight Loss	4	26.7%
Vomiting	4	26.7%
Jaundice	1	6.7%
Carcinoid Symptoms	3	20%

The ileum was the most common primary tumor site, found in seven patients (46.7%), followed by the jejunum in four patients (26.7%). The duodenum and appendix were each affected in two (13.3%) of cases as shown in Table 2.

**Table 2:** Primary Tumor Location in Stage IV SB-NET Patients (n=15) SB-NET: Small bowel neuroendocrine tumor

Primary Tumor Location	Number of Patients (n=15)	Percentage (%)
Duodenum	2	13.3%
Jejunum	4	26.7%
Ileum	7	46.7%
Appendix	2	13.3%

Seven patients (48%) had a Ki-67 index <2%, indicating a well-differentiated tumor with low proliferative activity. Moderate Ki-67 levels (3-20%) were observed in four (26%) cases, while another four patients (26%) had a high Ki-67 index (≥20%), suggesting a more aggressive disease course.

As shown in Table 3, all 15 patients underwent surgical resection of the primary tumor and hepatic metastasectomy. The most commonly performed procedure for primary tumor removal was small bowel resection in nine (60%) of the cases, followed by right hemicolectomy in four (26.7%) and Whipple’s procedure in two (13.3%) of the patients. For hepatic metastases, right lobe segmentectomy/metastasectomy was the most frequently done in four (26.7%), while other approaches, including bilateral metastasectomy/segmentectomy in three patients (20%) and non-anatomical liver resection in three patients (20%), were also utilized to reduce tumor burden.

**Table 3:** Surgical Procedures Performed in Stage IV SB-NET Patients (n=15) SB-NET: Small bowel neuroendocrine tumor

Surgical Procedure	Number of Patients (n=15)	Percentage (%)
Primary Tumor Resection		
Small Bowel Resection	9	60%
Right Hemicolectomy	4	26.7%
Whipple’s Procedure	2	13.3%
Hepatic Resection		
Left Lobe Metastasectomy/Segmentectomy	2	13.3%
Bilateral Metastasectomy/Segmentectomy	3	20%
Non-Anatomical Liver Resection	3	20%
Right Lobe Segmentectomy/Metastasectomy	4	26.7%
Right Lobectomy	3	20%

Adjuvant therapy was administered to all 15 patients. Octreotide alone was given to six (40%) patients, while chemotherapy alone was administered to five (33.3%) patients. The remaining four (26.7%) patients received a combination of octreotide and chemotherapy.

Postoperative 30-day mortality was one (6.7%) patient, while 90-day mortality was seen in two (13.3%) patients. Among patients who survived the immediate postoperative period, nine patients (60%) survived beyond five years, and five patients (33.3%) survived beyond 10 years as shown in Table 4.

**Table 4:** Survival and Mortality Outcomes

Survival Outcome	Value
30-day Mortality	1 (6.7%)
90-day Mortality	2 (13.3%)
5-Year Median Survival	9 (60%)
10-Year Median Survival	5 (33%)

An ANOVA test was conducted to compare the mean survival time across different Ki-67 index groups (<2%, 3-20%, and ≥20%) in patients with stage IV small bowel neuroendocrine tumors. The analysis yielded an F-statistic of 0.53 and a p-value of 0.60, indicating no statistically significant difference in survival time between the groups.

## Discussion

The management of neuroendocrine tumors (NETs) remains a complex challenge, particularly in LMICs, where resource limitations significantly impact diagnostic and treatment strategies [13]. In LMICs, delayed diagnosis is a recurring issue, often leading to more advanced disease at presentation [14]. This contrasts with high-income settings, where early detection and standardized management protocols contribute to better patient outcomes.

Compared to the study by Alfagih et al. at the Ottawa Hospital [15], our study showed a higher prevalence of abdominal pain (100%), whereas their study reported it in 46% of patients. Similarly, diarrhea was less frequent in our cohort (13.3%) compared to 24% in theirs. Carcinoid symptoms were reported in similar proportions (20% vs. 13%), while weight loss (26.7%) and vomiting (26.7%) were more prominent in our patients. In contrast, their study reported a higher prevalence of gastrointestinal (GI) bleeding (6%) and anemia (7%). These differences may reflect variations in disease progression at presentation, with patients in LMICs often experiencing diagnostic delays, a higher symptom burden, and an increased need for surgical intervention [16].

When comparing survival outcomes, our study demonstrated a median survival of 88 months (7.3 years) following loco-regional resection, whereas the study by Daskalakis et al. reported a median overall survival of 7.9 years (94.8 months) in patients who did not undergo immediate surgery [17]. These findings suggest that surgical intervention may not offer a significant survival advantage, as survival outcomes were similar between the groups.

The use of octreotide and chemotherapy in our cohort was aligned with global guidelines but was largely influenced by availability and financial constraints, particularly the lack of access to peptide receptor radionuclide therapy, which is widely used in high-income countries [18].

While our findings are consistent with existing literature regarding the predominance of SB-NETs and the role of surgery in treatment, the small sample size and single-institution nature of this study limit the generalizability of our results [19]. Larger, multicenter studies are needed to better evaluate long-term survival outcomes and the effectiveness of different therapeutic approaches. Further comparative research is essential to bridge the gap between LMICs and high-resource settings, ensuring that patients receive equitable and effective care regardless of healthcare system constraints. This report represents our institutional experience and serves as a foundation for future studies exploring the management of NETs in resource-limited environments.

## Conclusions

Our study provides insight into the survival outcomes of stage IV SB-NET patients undergoing loco-regional resection in a lower-middle-income setting. In our study, the absence of a control group limits definitive conclusions about the impact of surgical intervention on survival. While the findings suggest a potential survival benefit, they cannot be directly compared to non-surgical outcomes. These results align with existing literature emphasizing the need for a multidisciplinary approach that integrates surgery with systemic therapies. Given resource constraints in LMICs, individualized treatment strategies are crucial to optimizing patient outcomes.
